# Frequency and antibiogram of multi-drug resistant pseudomonas aeruginosa in a Tertiary Care Hospital of Pakistan

**DOI:** 10.12669/pjms.35.6.930

**Published:** 2019

**Authors:** Lubna Farooq, Zahida Memon, Muhammad Owais Ismail, Sara Sadiq

**Affiliations:** 1 Lubna Farooq, MBBS, M.Phil. Department of Pharmacology, Baqai Medical University, Karachi, Pakistan; 2 Zahida Memon, MBBS, M.Phil, PhD. Department of Pharmacology, Ziauddin University, Karachi, Pakistan; 3 Muhammad Owais Ismail, MBBS, M.Phil. Department of Pharmacology, Ziauddin University, Karachi, Pakistan; 4 Sara Sadiq, MBBS, M.Phil Department of Physiology, Ziauddin University, Karachi, Pakistan

**Keywords:** Pseudomonas aeruginosa, Multi-drug resistance, Ceftolozane/tazobactam

## Abstract

**Objective:**

To determine pathogen burden and susceptibility pattern of multi-drug resistant (MDR) *Pseudomonas aeruginosa* isolates from clinical specimens in Karachi.

**Methods:**

It was In-vitro Clinical study, conducted in department of Pharmacology, Ziauddin University, and isolates were collected from various specimens such as pus, tracheal aspiration, wound swab, blood and urine in Microbiology department of Ziauddin Hospital, Nazimabad campus, Karachi. The antibiotic susceptibility pattern was determined by Kirby Bauer Disc diffusion method. Samples were processed as per procedures defined by Clinical and Laboratory Standards Institute (CLSI) guidelines 2018.

**Results:**

About 55% were found to be multi drug resistant *P. aeruginosa*. Majority of the isolates (35.4%) were recovered from the age range 60-80 years. Maximum number of MDR *P. aeruginosa* was isolated from pus (33.1%) followed by tracheal aspiration (20.6%). Highest sensitivity was seen by colistin (100%) followed by ceftolozane/tazobactam (60%). Least sensitivity was observed with imipenem (19%). However, increase trend of resistance was seen among all antipesudomonal drugs.

**Conclusion:**

Increasing frequency of infections due to MDR *P. aeruginosa* is an emerging threat in our set up which can be prevented by prescribing antibiotics judiciously. Consistent lab detection and surveillance regarding this resistant pathogen is compulsory for providing effective health care to community.

## INTRODUCTION

Antimicrobial agents are the main therapeutic tool in medicine to treat variety of infections caused by bacteria. The development of antibiotics is considered as one of the most important advances in modern science. Antibiotics have saved millions of lives. Emergence of resistance against antimicrobials are one of the most important threats globally.^1^ The heightened use and sometimes misuse of antibiotics results in emergence of bacteria’s that don’t respond to therapy anymore.^2^

*Pseudomonas aeruginosa* is aerobic, non-fermenting Gram-negative bacilli, which is most commonly involved in opportunistic infections mostly in the nosocomial setting.^3^ *P.aeruginosa* is playing havoc on medical therapeutics and has ability to acquire and express multiple resistance mechanisms mediated by loss of the OprD porins deletion, overexpression of efflux pumps, modification in target site and production of certain b-lactamases and carbapenamases enzymes.^4^

Currently available drugs against *P.aeruginosa* include fluoroquinolones (ofloxacin, ciprofloxacin), antipseudomonal penicillins (ticarcalin, piperacllin), cephalosporins (ceftazidime, cefepime), aminoglycosides (amikacin, gentamicin) and carbapenems (imipenem, meropenum).^5^ Multidrug resistance (MDR) P.aeruginosa was defined as “acquired non-susceptibility to at least one agent in three or more antipseudomonal classes (carbapenems, fluoroquinolones, penicillins, cephalosporins and aminoglycosides).^6^

MDR *P. aeruginosa* is cosmopolitan superbug which has been associated with adverse clinical outcomes, including increased mortality and morbidity.^7^ Mortality rate has been reported up to 20% and about 10,000 patients are admitted per year due to infections caused by MDR *P*. *aeruginosa*.^8^ The Infectious Data presented by the Center for Disease Control and Prevention (CDC) in the USA revealed that MDR *P. aeruginosa* caused diverse variety of infections and was found to be one of the most common causes of nosocomial pneumonia, urinary tract infections, eye and ear infections, bacteremia and surgical site infections.^8^

Concurrently, the overuse and irrational use of antibiotics and de novo emergence of resistance in a specific bacteria have resulted in the emergence of drug resistant bacteria.^9^ This makes *P.aeruginosa* virtually invincible against many antibiotics for treatment of life threatening infections.^10^

Current studies on antimicrobial resistance pattern of MDR *P. aeruginosa* are necessary to evaluate sensitivity pattern of this organism against usually prescribed antibiotics agents. This would help the physicians to optimize the current effective treatment options.

The present study was conducted to evaluate the frequency and antibiotic susceptibility trends of MDR *P. aeruginosa* isolated from different clinical samples in hospital of Karachi.

## METHODS

It was an in vitro clinical trial. The study was conducted in Department of Pharmacology, Ziauddin University and the samples were collected from Ziauddin hospital, Nazimabad. Ethical approval (Ref.no. 0250917LJPHARM) for the study was obtained from the hospitals ethics committee on September 13, 2017. A total of 1900 specimens of pus, wound swabs, blood, urine, endotracheal secretions were processed for culture and sensitivity as per defined guide lines in Microbiology lab of Ziauddin hospital, Nazimabad from October 2017 to April 2018.

Specimens were inoculated on routine culture media on MacConkey agar (Oxoid) and Blood agar (Oxoid). The plates were incubated at 37^o^C for 24 hrs. All gram negative, catalase and oxidase positive colonies were identified up to species level by standard microbiological procedure.

Antibiotic susceptibility was checked by *Kirby-Bauer’s* disc diffusion method. In this method a lawn of bacterial inoculum was made on 150 mm Mueller Hinton Agar plate (Oxoid UK). Antibiotic disc of Piperacillin/ tazobactam (100/10ugm), Imipenem (10μgm), Aztreonam (30 μgm), Ceftazidime (30μgm), Amikacin (30μgm), Gentamicin (10μgm), Ciprofloxacin (5μgm), Colistin (10μgm), Ceftolozane/tazobactam (30/10μgm) were placed on agar plate. Before determination of results, plates were incubated for 16-24 h at 35^o^C. The zones of growth inhibition around each of the antibiotic disc were measured in accordance to CLSI guidelines (2018) and labeled as either sensitive or resistant.^11^

Data was analyzed by using Statistical Package for Social Sciences (SPSS) version 21. Descriptive analyses for numerical variables were mentioned as Mean with standard deviation. Frequencies and percentages were calculated for categorical variables. Chi square test was applied to measure the association between sensitivity and resistance patterns of drugs. P value of less than 0.05 was considered as significant.

## RESULTS

On the basis of identification methods, one hundred and seventy-six (176) strains of *P. aeruginosa* were isolated from 1900 specimen. Out of which 97(55%) were MDR *P.aeruginosa* and 79 (45%) were non MDR *P.aeruginosa. Looking over the gender wise frequency, the* MDR *P. aeruginosa* were predominant in females that were 54% as compared to males, which was 46% as shown in Table-I.

**Table I:** Total samples of *P.aeruginosa*.

Total samples		MDR	Non MDR
	1900	97 (55%)	79 (45%)
Male	85	45 (46%)	40 (50.6 %)
Female	89	52 (54%)	37 (46 .8%)

Most of the isolates were obtained from pus (34%) followed by tracheal aspiration (20.6%) then from urine 18.6% and least were obtained from ear swab 2.1% as shown in Table-II. P value was less than 0.05, which was statistically significant.

**Table II:** Frequency of MDR *P. aeruginosa* in specimen.

Source	MDR 97 (55 %)	Non MDR 79 (45 %)	P value
Pus	33 (34.0%)	12 (15.1%)	0.024
Tracheal asp	20 (20.6%)	17 (21.5%)	
Urine	18 (18.6%)	24 (30.4%)	
Sputum	14 (14.4%)	14 (17.7%)	
Blood	10 (10.1%)	8 (10.1%)	
Ear swab	2 (2.1%)	4 (5.1%)	

The organism was predominantly isolated in Indoor patient department as compared to outpatient department, which was 59% and 41% respectively.

In wards majority of the MDR *P .aeruginosa* were isolated from surgical ward 26(27.1%), and least from Gynecology ward 3(4.1%), as shown in Table-III.

**Table III:** Percentage of MDR isolates in different Departments.

Department	MDR	Non MDR
Gynecology ward	3 (4.1%)	0 (0%)
ICU	16 (16.5%)	17 (21.5%)
Surgical ward	26 (27.1%)	30 (38.0%)
Medicine ward	11 (11.3%)	8 (10.1%)

**Fig. 1:**
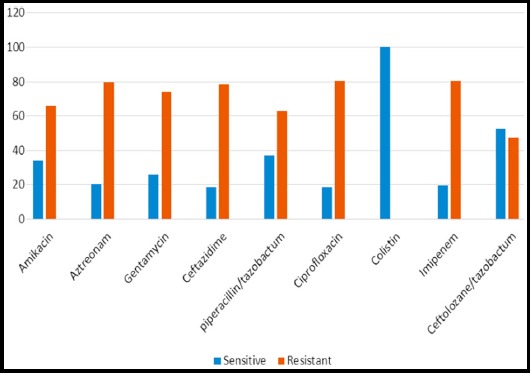
Sensitivity and resistance pattern of *P. aeruginosa* (MDR).

MDR *P. aeruginosa* showed increase resistance to almost all antipseudomonal drugs. Highest resistance was observed with imipenem (81.6%). The resistance pattern against Ciprofloxacin (80.4%), Ceftazidime (78%), Gentamycin (74.2%), Amikacin (66%), Piperacllin /tazobactam (62%) & Ceftolozane/tazobactam (40%) respectively. Colistin was 100% sensitive to all isolates of MDR *P. aeruginosa* as shown in Fig. 1.

## DISCUSSION

*P. aeruginosa* is a notorious Gram negative bacillus that is associated with many diseases such as pneumonia, bacteremia, urinary tract, skin and soft tissues infections etc. especially in immunocompromised patients.^12^ Clinical isolates of *P. aeruginosa* may demonstrate resistance to multiple classes of antibiotics leaving Clinicians with few therapeutic antibacterial drugs or their regimen options from which to choose for treatment of infectious diseases.

The frequency of MDR *P. aeruginosa* in our set up was found to be 55.1%, while 57.8% were reported by Ameen et al in Karachi 2015.^13^ Studies done in Lahore, Punjab and Rawalpindi revealed following results 22.7%, 20% and 21%.^14^-^16^ World wide data reported 85% of prevalence by a study conducted in tertiary care hospital of India.^17^ In Africa prevalence was found to be 47.8% in 2017.^18^ However higher frequency (56%) was also recorded in Egypt in 2015.^19^ In 2010, a study conducted in Taxes reported 14% of MDR isolates from 235 strains of pseudomonas.^20^ Keeping in view the above extensive literature survey, it can be stated that resistance against *P. aeruginosa* has been gradually increasing with the passage of time in all parts of the world including Pakistan. This increase in resistance against this organism is possibly due to its peculiar structure that *P. aeruginosa* has a large genome which contain 6.3 million base pairs and this sequence is considered to be the largest among all bacteria. This versatility in its sequence is responsible for producing intrinsic resistance to antimicrobials and also contain highest number of regulatory genes which are responsible for either mutational change in efflux pump and/or in porins structure.^21^

In our study MDR *P. aeruginosa* was predominant in females (54%) as compared to males (46%).Almost similar results were reported in a study done in Nepal which showed following results females (55.1%) & males (44.9%).^22^ Contrasting results were found in a study done in Pakistan in 2017 showed MDR *P.aeruginosa* was more predominant in males (55%) than in females (45%).^23^ Studies done in India and Iraq also showed contrasting results. In these studies, MDR *P. aeruginosa* was more ubiquitous in males as compared to females that was (55%) and (56%) respectively.^3^ This might be explained by the fact that gender prevalence may also differ with geographical variation and study period.

Majority of *P.aeruginosa* isolates in our study were recovered from pus (33.1%) followed by tracheal aspiration (20.6%) and urine (18.6%).Our results concur to some extent with earlier studies where pus samples were most common source.^16^ The justification of presence of highest number of isolates in pus is due to the fact that majority of patients had postoperative wound complications and these wound sites are easy targets for nosocomial pathogens. Inadequate antiseptic measures and poor hygiene in wards are other possible contributory factors in acquiring the resistant strains.

In our study major contribution of MDR strains was from surgical ward (26.8%) followed by ICU (16.5%), medicine ward (11.3%) and gynecological ward (4.1%). A study done by Saeed WM et al in 2018 has linked ICU significantly to be the major source of MDR isolates. Intensive care unit patients especially create an environment for infection because of the debilitating effect of a prolonged hospitalization and the application of medical equipment’s. (Airways, cannula, catheters etc.).^16^

Currently available drugs against MDR *P. aeruginosa* include Fluoroquinolones (ofloxacin, ciprofloxacin) antipseudomonal penicillins (ticarcalin, piperacllin), cephalosporins (ceftazidime, cefepime), aminoglycosides (amikacin, gentamicin) and carbapenems (Imipenem, meropenum). However, due to resistance strains of *P.aeruginosa* *has* finally outsmarted our best treatment options. Like other studies our research has also demonstrated high resistance against all beta-lactam antibiotics.^24^

Regarding antibiotic susceptibility, the highest resistance of MDR strains was found to imipenem (81.6%), followed by Ciprofloxacin (80.4%), ceftazidime (78%), gentamycin (74.2%) while these strains showed the highest susceptibility to Colistin (100%) and C/T (40%).

Ameen et al, reported highest resistance of MDR P.aeruginosa against imipenem (100%) followed by gentamycin (98%), amikacin (77.8%), piperacillin/tazobactam (68.1%).^13^ It is evident that MDR strains of *pseudomonas* are accelerating in Pakistan. This increase in resistance in our community is due to the fact that these drugs are widely prescribed in secondary and tertiary care hospitals. In accordance to accepted selected theory there is causal relationship between antimicrobials use and development of resistance. In contrast, studies done in India reported that imipenem was 100% sensitive to *P. aeruginosa* followed by piperacllin/tazobactum 72%.^9^ A study done in Iraq showed that ceftazidime was 100% resistant whereas imipenem 95% sensitive to *P.aeruginosa*.^3^ The high sensitivity against imipenem can be attributed because of low exposure and limited use of this drug in their hospitals. In our study, Colistin (Polymyxin B) was 100% sensitive to MDR strains. This finding was similar to another study done in Pakistan which showed that Colistin was most sensitive drug among all antibiotics. In another study conducted in Europe, Sader et al, found Colistin to be the most effective in vitro, against MDR *P. aeruginosa*. Colistin is Polymyxin B antibiotic which is used for gram negative bacteria. Colistin is sensitive drug in our setup and also around the globe but clinical use of this drug as an empirical therapy is limited because of its narrow therapeutic index and significant side effects.^25^

### Limitations of the study

This study was conducted in only one center in Karachi. It is strongly recommended that this research must be done at lager scale and must involve other clinical settings of country to obtain more valid antibiotic susceptibility pattern against MDR *P .aeruginosa*, which will help in the controlling the spread of infections caused by this lethal organism and will also be useful for better management of infectious diseases.

## CONCLUSION

The resistance of *P. aeruginosa* was amplified over a past few decades. Present study showed high resistance of MDR strains against commonly used therapeutic agents. Ceftolozane/tazobactum is the drug that showed best activity against Pseudomonas. Keeping in view above discussion, it can be recommended that the therapeutic use of broad spectrum antibiotics should be reserved only for severe and life threatening infections. Consistent lab detection and surveillance regarding this resistant pathogen is compulsory for providing effective health care to community.

### Authors’ Contribution:

**LF:** Conceived, designed and did statistical analysis & editing of manuscript.

**LF, MOI & SS:** Did data collection and manuscript writing.

**ZM:** Did review and final approval of manuscript.
